# A comparison of thyroidal protection by stable iodine or perchlorate in the case of acute or prolonged radioiodine exposure

**DOI:** 10.1007/s00204-020-02809-z

**Published:** 2020-07-12

**Authors:** Stefan Eder, Cornelius Hermann, Andreas Lamkowski, Manabu Kinoshita, Tetsuo Yamamoto, Michael Abend, Nariyoshi Shinomiya, Matthias Port, Alexis Rump

**Affiliations:** 1 Bundeswehr Institute of Radiobiology, Neuherberg Str. 11, 80937 Munich, Germany; 2 Japan Self Defense Forces National Defense Medical College Research Institute, Tokorozawa, Japan; 3 Japan Ground Self Defense Forces Military Medicine Research Unit and Ministry of Defense Clinic, Tokyo, Japan

**Keywords:** Medical NRBC protection, Nuclear and radiological emergency, Radioiodine, Iodine blockade, Perchlorate

## Abstract

In the case of a nuclear power plant accident, repetitive/prolonged radioiodine release may occur. Radioiodine accumulates in the thyroid and by irradiation enhances the risk of cancer. Large doses of non-radioactive iodine may protect the thyroid by inhibiting radioiodine uptake into the gland (iodine blockade). Protection is based on a competition at the active carrier site in the cellular membrane and the Wolff–Chaikoff effect, the latter being, however, only transient (24–48 h). Perchlorate may alternatively provide protection by a carrier competition mechanism only. Perchlorate has, however, a stronger affinity to the carrier than iodide. Based on an established biokinetic–dosimetric model developed to study iodine blockade, and after its extension to describe perchlorate pharmacokinetics and the inhibition of iodine transport through the carrier, we computed the protective efficacies that can be achieved by stable iodine or perchlorate in the case of an acute or prolonged radioiodine exposure. In the case of acute radioiodine exposure, perchlorate is less potent than stable iodine considering its ED_50._ A dose of 100 mg stable iodine has roughly the same protective efficacy as 1000 mg perchlorate. For prolonged exposures, single doses of protective agents, whether stable iodine or perchlorate, offer substantially lower protection than after acute radioiodine exposure, and thus repetitive administrations seem necessary. In case of prolonged exposure, the higher affinity of perchlorate for the carrier in combination with the fading Wolff–Chaikoff effect of iodine confers perchlorate a higher protective efficacy compared to stable iodine. Taking into account the frequency and seriousness of adverse effects, iodine and perchlorate at equieffective dosages seem to be alternatives in case of short-term acute radioiodine exposure, whereas preference should be given to perchlorate in view of its higher protective efficacy in the case of longer lasting radioiodine exposures.

## Introduction

Large amounts of radioactive materials may be released in the case of a nuclear emergency, including fission products, activation products, uranium, plutonium, and transuranic elements (Wohni 1995). The precise isotopic composition of the released radioactive material may greatly differ in the case of accidents in nuclear facilities and the resulting fallout will also depend on the meteorological conditions and the distance from the source (Wohni 1995; Imanaka et al. 2015). In particular, volatile radionuclides like radioiodine or cesium may reach higher atmospheric levels and be transported over larger distances compared to fuel particles that may be expected to deposit in the near zone around a plant, as also happened in Chernobyl (Wohni 1995).

Among the fission products formed in a nuclear power reactor, radioiodine is one nuclide of particular concern. Uranium-235 usually splits asymmetrically and radioiodine (I-131) falls in one of the favored mass number regions of the fission products (bimodal distribution with peaks between 90–100 and 130–140). Moreover, the fission yield of iodine-131 is relatively high with about 3% (the yield is the number of a particular radionuclide produced for every 100 fission events), and because of its high volatility, radioiodine is readily spread in different physical forms like gaseous inorganic, gaseous organic iodine, or adsorbed to particles (National Cancer Institute 2015; Chabot 2016).

Iodine is rapidly absorbed into the blood by inhalation or ingestion and, after distribution in the extracellular space, it is transported and concentrated in the thyroid gland through an active carrier mechanism (sodium iodide symporter, abbreviated as NI-symporter) in the basolateral membrane of the follicular cells (Wolff 1998). The uptake fraction into the gland in healthy people amounts from 10 to 40% of the intake into the body and about half of this amount enters the gland within 3–6.5 h (Kovari 1994; Geoffroy et al. 2000; Verger et al. 2001). Thyroidal iodine uptake heavily depends on nutritional iodine supply (Takamura et al. 2004; Reiners et al. 2013) and the function of the gland (e.g., iodide uptake fraction up to 80% in patients with Grave’s hyperthyroidism) (Horn-Lodewyk 2019). In the case of radioiodine uptake, this leads to an irradiation of the tissue, which may cause destruction and hypothyroidism at very high activities, or stochastic damages with an increased incidence of thyroid cancers on the long run. This occurred following the Chernobyl accident with an increase in the frequency of thyroid cancers among the population who had been exposed to radioiodine as children (Lomat et al. 1997; Henriksen et al. 2014). The elimination of iodine that has not been taken up into the gland out of the body occurs through the kidney with a clearance between 30 and 50 ml/min (Geoffroy et al. 2000).

A reduction of radioiodine uptake into the thyroid and a protection of the gland can be achieved by administering a large dose of stable (not radioactive) iodine shortly before or shortly after radioiodine exposure (“iodine blockade”) (Geoffroy et al. 2000; Verger et al. 2001; Rump et al. 2016; WHO 2017) and “provision of iodine thyroid blocking (ITB) to people who are at risk of being exposed to radioiodine should be implemented as an urgent protective action” according to the World Health Organization (WHO 2017). Iodine blockade in the case of radioiodine exposure is also recommended by national guidelines in many countries (e.g., in Germany or France) (ASN 2008; SSK 2018).

As a general rule, a single administration of stable iodine is recommended, although it is acknowledged that repetitive administrations might be required in the case of prolonged exposures (WHO 2017). Guidelines diverge on this point and there are no clear cut rules (Jourdain et al. 2010). In Chernobyl as well as in Fukushima radioactive material was released repetitively over 7–10 days. In Fukushima, the total amount of released radioactivity was, nevertheless, far less than in Chernobyl and the radionuclide mixtures also differed. Moreover, the time-course of radioactivity release was different: Chernobyl was a power surge accident caused by a failure to control the fission chain reaction and led to an instantaneous explosion of the reactor and destruction of the building. Radioactivity release was most important the first day (April 26, 1986: > 2000 PBq day^−1^) and far less in the further course (roughly between 200 and 500 PBq day^−1^) (Imanaka et al. 2015). However, the time-course may show a different pattern depending on measurement location and meteorological conditions (e.g., I-131 and Cs-137 air concentration showed two peaks measured at Munich–Neuherberg) (Winkelmann 1986; UNSCEAR 1988). Fukushima was a loss-of-coolant accident and the decay heat from fission led to the meltdown of the reactor cores. Containment vessels were vented to reduce pressure and several hydrogen explosions occurred and damaged the buildings. The time-course was, however, different in the three damaged reactor units and the pattern of radioactivity release irregular (peak on March 15, 2011: 25–30 PBq day^−1^ followed by fluctuations between 5 and 10 Bq day^−1^ and a second peak on March 23 between 15 and 20 Bq day^−1^ and a decrease thereafter) (Imanaka et al. 2015). Thus, in the case of a power plant accident, a repetitive or continuous exposure to radioiodine must be expected, although the time-course patterns may substantially differ, and guiding principles on repetitive iodine administration should be worked out for physicians and authorities. Moreover, the question seems legitimate whether other means than stable iodine for protecting the gland against radioiodine are available and may present advantages. This issue requires considering the mechanism of action of large doses of stable iodine.

Several mechanisms are involved in thyroidal protection: 1. Competition between radioiodine and stable iodine at the membrane NI-symporter site of the thyroid cells and 2. A rapid inhibition of hormonal synthesis, i.e., an inhibition of the inclusion of (radio)iodine into the hormone precursor thyroglobuline (Wolff–Chaikoff effect) (Wolff et al. 1948; Geoffroy et al. 2000; Verger et al. 2001). The mechanism of the latter is not fully understood. It seems that it is at least in part mediated by the inhibition of peroxidase activity in the follicular cells and this effect is fading after 24–48 h (Geoffroy et al. 2000; Verger et al. 2001; Leung et al. 2014). Therefore, protection provided by the Wolff–Chaikoff effect may be expected to become ineffective in the case of prolonged radioiodine exposure.

An alternative could be perchlorate (Harris et al. 2009). The NI-symporter is not selective for iodide, but transports several related monovalent anions. Affinity of the carrier decreases in the same order as the Hofmeister or lyotropic series: TcO_4_ > ClO_4_ > ReO_4_ > SCN > BF_4_ > I > NO_3_ > Br > Cl (Wolff 1964, 1998). Thus, perchlorate has even a higher affinity to the NI-symporter than iodide. On the other side, perchlorate has been reported to have no effect on the organification of iodine, i.e., there is no saturation mechanism comparable to the Wolff–Chaikoff effect (Wolff 1998). Thus, as a consequence of a single and simple competition mechanism at the carrier site that is not complicated by effects on later metabolic steps, theoretically, thyroidal protection may possibly be less prone to variability and easier to predict as in the case of iodine. Perchlorate can also cause an iodide discharge out of the thyroid. This is used in the iodide-perchlorate discharge test for the diagnosis of the Pendred syndrome, an autosomal recessive disorder associated with an impairment of the pendrin-mediated transport of iodide through the apical membrane of the thyrocytes into the follicular lumen and, thus, leading to an iodide organification defect (Reardon et al. 1996; Kopp et al. 2011). This discharge effect of perchlorate could also possibly contribute to thyroid protection against radioiodine. Like iodine, perchlorate is extensively (95%) and rapidly absorbed from the gastrointestinal tract (perchlorate is present in urine from 10 min after ingestion) in humans (ATDSR 2008; BAuA 2016). It is eliminated virtually unchanged through the kidney with a half-time ranging from below 8 h to 12 h in humans (ATDSR 2008; Lorber 2009; BAuA 2016).

Based on an established biokinetic-dosimetric model developed to study iodine blockade (Rump et al. 2019), and after its extension to describe perchlorate pharmacokinetics and its inhibition of iodine transport through the NI-symporter, we calculated protective efficacies that can be achieved by stable iodine or perchlorate in the case of an acute or prolonged radioiodine exposure. The objective of the simulations was to determine which of both agents might be preferable at what dosages in different scenarios.

## Method

### Pharmacokinetic models for iodine and perchlorate

**Fig. 1:**
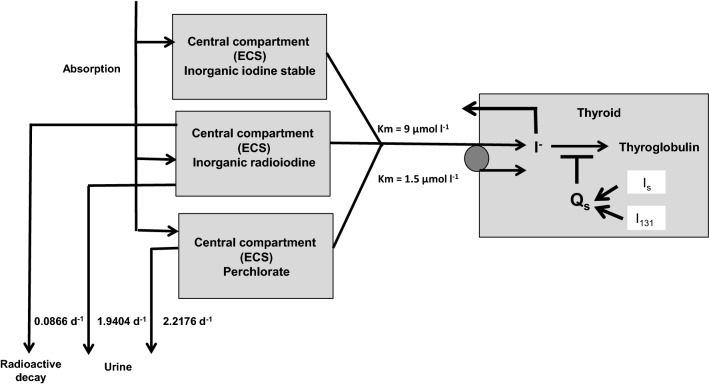
Compartment models for radioiodine and perchlorate with an integrated carrier uptake mechanism described by a Michaelis–Menten kinetic for thyroidal iodine uptake. The competition of perchlorate and radioiodine at the carrier site is modeled by applying the rate law for monomolecular irreversible enzyme reactions to the transport mechanism. The Wolff–Chaikoff effect is modeled by a total thyroidal uptake block for iodine (lasting 24–48 h), starting when the gland is saturated (uptake amount *Q*_s_ 350 µg iodine). Although the model for stable iodine and perchlorate is shown in the same figure, the protective efficacy of both agents is simulated separately

The biokinetics of iodine was described as previously reported by a simple two-compartment model (Rump et al. 2019) derived from the iodine model introduced by Riggs (1952) and widely used in radiation protection by the International Commission for Radioprotection (ICRP) (ICRP 1994, 1997) (Fig. 1).

The central compartment represents the extracellular space including red blood cells with an estimated approximate volume of distribution of 16 l (water: 60% of body weight; extracellular space 1/3, i.e., 14 l and red blood cells about 2 l). As we are interested in the competition of radioiodine and stable iodine, we did not consider the kinetics of iodine integrated with thyroid hormones being secreted from the thyroid and their elimination, and therefore, the corresponding compartments were deleted.

Iodine is eliminated from the central compartment by renal excretion that is a first-order process that is not saturable (rate constant: 1.9404 day^−1^). In the case of radioiodine only, an additional elimination out of the central compartment occurs by physical decay (decay constant 0.086625 day^−1^ corresponding to a half-life of 8 days for I-131 and 1.2669 day^−1^ corresponding to a half-life of 13.13 h for I-123) (Health Physics Society n.d.). In addition, radioiodine as well as stable iodine are transported into the peripheral compartment representing the thyroid and acting as a sink.

As iodine transport into the thyroid occurs through an active transport (NI-symporter) and not by passive diffusion, a carrier mechanism was integrated with the model This transfer process can be described in analogy to an enzyme reaction by Michaelis–Menten kinetics:

$$ T = \left( {T_{\max } *C} \right)/\left( {K_{m} + C} \right) $$

For the human NI-symporter, a *K*_*m*_ of 9 µmol/l has been reported and this value was used in our model (Darrouzet et al. 2014). *T*_max_ was derived from the *K*_*m*_ and the constant rate k of the ICRP model applicable when the transport process approaches a first-order kinetic at very low iodide concentration (*k* = 0.8316 day^−1^): *T*_max_ = *K*_*m*_ * *k* = 9 * 0.8316 = 7.48 µmol l^−1^ day^−1^.

The biokinetics of perchlorate is described by a simple one-compartment model as described by Lorber (2009) (the second compartment representing the bladder is not taken into account in our model). The renal elimination of perchlorate out of the body follows first-order kinetics and we used the parameters reported in the literature (elimination rate constant 0.0924 h^−1^, half-life 7.5 h; volume of distribution 0.34 l kg^−1^, i.e., for a 70 kg adult 23.8 l) (Crump et al. 2005; Lorber 2009) (Fig. 1).

### Modeling of radioiodine uptake inhibition by stable iodine or perchlorate

As reported previously (Rump et al. 2019), we applied the rate law for monomolecular irreversible enzyme reactions with any number i of competing substrates to the carrier-mediated transport process (Chou et al. 1977; Schäuble et al. 2013):

$$T1= \frac{T\mathrm{m}\mathrm{a}\mathrm{x}*C1}{Km1*\left(1+\sum_{i=2}^{n}\frac{Ci}{Kmi} \right)+C1}$$

with *T*_*i*_ the transport rate for substrate *i*, *C*_*i*_ the concentration of substrate *i*, *K*_*mi*_ the Michaelis–Menten constant for substrate *i*, and *T*_max_ the maximum transport rate.

In the case of iodine blockade, the two competing entities are chemically identical (radioiodine and stable iodine) and share the same Michaelis–Menten constant (*K*_*m*_ = 9 µmol l^−1^). For perchlorate, the Michaelis–Menten constant has been given with 1.5 µmol l^−1^ (Kosugi et al. 1996), but a revised lower value of 0.59 µmol l^−1^ was reported recently with the indication that the latter was better suited to describe the kinetics of lower dose levels below 100 µg kg^−1^ day^−1^ (Schlosser 2016). When validating our model using empirical data, we used both constants to identify the value actually better suited to study protective effects against radioiodine exposure.

The pharmacokinetic model for iodine presented in the previous paragraph takes into account only protective effects by competition at the NI-symporter site. High doses of iodine, however, are known to temporarily block the thyroid uptake of iodine completely (Wolff-Chaikoff effect). As described previously, we modeled this blockade by adding an additional saturation mechanism in our pharmacokinetic iodine model (Rump et al. 2019) (Fig. 1). This total thyroidal uptake block becomes effective when the iodine content of the gland increases by 0.35 mg (2.7581 µmol) (Ramsden et al. 1967; Wootton et al. 1978). In our simulations, we varied the time the Wolff–Chaikoff effect remains active between 24 and 48 h, as described in the literature (Leung et al. 2014).

### Calculation of the thyroid equivalent dose from the radioiodine uptake into the gland

A simple calculation of the (committed effective) dose by multiplication of the radioiodine intake with the dose coefficient for inhalation or ingestion of radioiodine is not possible in our case, as it supposes a defined and constant uptake fraction into the thyroid. Therefore, we used the Marinelli/Quimby method (1948) for the contribution of the ß-radiation and the geometrical factor method of Hine et al. (1956) for the (low) contribution of the ɣ radiation to the thyroid equivalent dose. The method has also been described for thyroid equivalent dose computations in the more recent literature (Stabin et al. 2006; Spetz 2010; National Cancer Institute 2015). As radioiodine mostly concentrates in the gland, for radiation emitted from other source organs to the thyroid as a target, the specific absorbed fractions were set to 0. Thus, the following equation was used:

$$  D = C_{\max } * \, T_{{{\text{eff}}}} * \, \left( {73.8 \, * \, _{{\overline{E}} {\beta }} + \, 0.0346 \, * \, T \, * \, \overline{g}} \right) $$

With *D* the total dose from *ß* and *ɣ* radiation (rad), *C*_max_ the maximum concentration of the radionuclide in tissue (µCi g^−1^), *T*_eff_ the effective half-life in the tissue (days) (7.3 days for I-131 in adults), *Ē*_*ß*_ the average beta energy (MeV per disintegration) (0.18 MeV for I-131), *Ƭ* the specific *ɣ* ray constant (*R* per mCi h^−1^ at 1 cm)(2.2 *R* mCi^−1^ h^−1^), and *ḡ* the average geometrical factor for the tissue or organ, equal to 3 π *r* for spheres with radii < 10 cm) (*r* = 1.27 cm in adults assuming that the thyroid is made of two identical spheres of unit density) (values from National Cancer Institute 2015). As we used radioiodine amounts and not concentrations in our model, we replaced the maximum concentration *C*_max_ by the maximum accumulated amount divided by the thyroid weight assumed to be on the average 17 g in an adult (National Cancer Institute 2015). After transformation taking into account the units used in the original equation, we applied the following formula to calculate the thyroid equivalent dose:

$$ D \, \left( {{\text{mSv}}} \right) \, = { 1}.{647 }*{ 1}0^{{ - {3}}} *{\text{ accumulated I}} - {\text{131 in the thyroid }}\left( {{\text{Bq}}} \right) $$

It should be emphasized that the equation is used to calculate the thyroid equivalent dose (energy dose * quality factor of the radiation, for *ß* and *ɣ* radiation the quality factor = 1), and not an effective dose additionally taking into account the radiation sensitivity of a tissue regarding stochastic health effects (effective dose = equivalent dose * sensitivity factor, for the thyroid the sensitivity factor = 0.05, but equivalent and effective doses are both expressed with the same unit Sv).

### Validation of the models

The iodine blockade model was previously validated by comparison with results obtained by the Integrated Modules for Bioassay Analysis (IMBA) software (Rump et al. 2019).

The pharmacokinetic model for perchlorate was applied to experimental data reported in the literature for volunteers or workers professionally exposed to perchlorate (Lawrence et al. 2000; Braverman et al. 2005; Lorber 2009). The administered single doses or the continuous exposure data were entered in our model and the serum concentrations calculated were compared to the measured values at defined time points.

In addition, we used the complete combined perchlorate-radioiodine model with competition at the carrier site to compute the protective efficacy after the experimental administration of radioiodine (iodine-123) to volunteers as described in several studies (Lawrence et al. 2000; Greer et al. 2002; Bravermann et al. 2005; Hänscheid et al. 2011) and compared our results with the measured values reported. Computations were performed for Michaelis–Menten constants for perchlorate described in the literature: 1.5 µmol l^−1^ (Kosugi et al. 1996) or 0.59 µmol l^−1^, as recently reported for lower dosages up to 100 µg kg^−1^ day^−1^ (Schlosser 2016).

The protective efficacy was determined as the complementary value of the quotient of the thyroidal radioiodine dose uptake fraction with and without perchlorate administration:

$${\text{Efficacy }} = { 1 }{-} \, \left( {{\text{thyroidal iodine uptake fraction with perchlorate blockade}}/{\text{uptake fraction without perchlorate}}} \right)$$

In our simulations, we added radioiodine (in this case iodine-123) as well as perchlorate directly into the central compartment, as for an administration by injection, to avoid possible effects related to the structure and parameters of the inhalational or gastrointestinal models. This seems legitimate as the absorption rates for both, iodine and perchlorate, are rapid and the extent almost complete (Geoffroy et al. 2000; ATDSR 2008; BAuA 2016).

### Comparison of the median protective doses of perchlorate and stable iodine.

We determined the equivalent thyroid dose resulting from an acute single intake of radioiodine (in this case iodine-131) (700,000 Bq, leading roughly to the thyroid dose limit of 300 mSv according to German regulations; this activity corresponds to a molar amount of 1.16.10^–6^ µmol; specific activity of iodine-131: 4.6 * 10^15^ Bq/g) and added different doses of perchlorate or stable iodine in a range of 1–1000 mg, administered at the same time as radioiodine. Again, iodine-131 as well as perchlorate were entered directly into the central compartment.

The efficacy was based on iodine-131 accumulated up to 24 h after exposure as described in the previous section.

The dose–effect relation was examined by fitting the data to a sigmoidal Hill equation with three parameters $(\mathrm{E}=\frac{a*{D}^{b}}{{D50}^{b}+ {D}^{b}})$. For comparison to the protective efficacy of perchlorate, we used data previously determined using the same method for stable iodine (Rump et al. 2019).

### Estimation of the protective efficacy of perchlorate or stable iodine depending on the time of blockade initiation

In a further simulation, we determined the protective efficacy of single doses of 100 mg or 1000 mg perchlorate or 100 mg stable iodine administered at different time points ranging from 72 h before to 24 h after acute iodine-131 exposure. Acute iodine-131 exposure again amounted to 700,000 Bq entered into the central compartment at *t* = 0. Protective efficacy was calculated based on iodine-131 accumulated up to 24 h after exposure.

For the iodine blockade, protective efficacy was determined with or without taking into account the Wolff–Chaikoff effect as described in a previous section. We considered the transient effect to last between 24 and 48 h, as mostly described in the literature (Leung et al. 2014). Only for illustration, we also simulated protective efficacy with a Wolff-Chaikoff effect being effective to infinity, although this is not a realistic assumption.

### Protective efficacy of repetitive doses of perchlorate or stable iodine after acute iodine-131 exposure

Calculations were performed for an acute intake of 700,000 Bq iodine-131 into the central compartment. Doses of perchlorate or stable iodine ranging from 100 to 1000 mg were administered once simultaneously to iodine-131 at *t* = 0 and protective efficacy was compared to a dosage regimen splitting the dose into two individual doses given at an interval of 12 h; the first dose being also administered at *t* = 0. Protective efficacy was calculated based on iodine-131 accumulated up to 24 h after exposure as previously.

### Protective efficacy of perchlorate or stable iodine in the case of continuous iodine-131 exposure

Radioactivity release lasted over several days after the reactor accidents of Chernobyl and Fukushima (Imanaka et al. 2015). A group of 45 evacuees or short-term visitors who had stayed in Fukushima from March 11 to 18, 2011 was examined in a whole-body counter at Nagasaki University. The mean iodine-131 activity measured was 574.7 Bq and the maximum activity amounted to 3940 Bq (Matsuda et al. 2013). Based on the latter maximum activity, we determined that this would result from a continuous intake of 1868 Bq/day leading to a thyroid equivalent dose of 6.25 mSv based on iodine-131 accumulated up to 24 h after the end of iodine-131 exposure. Although this low dose would not be an indication for a blockade of the gland, we simulated the effects that single or repetitive daily doses of perchlorate or stable iodine would have had on the thyroid equivalent dose. In the case of iodine blockade, the duration of the Wolff–Chaikoff effect was varied from 24 to 48 h.

## Results

### Validation of the perchlorate model

**Fig. 2:**
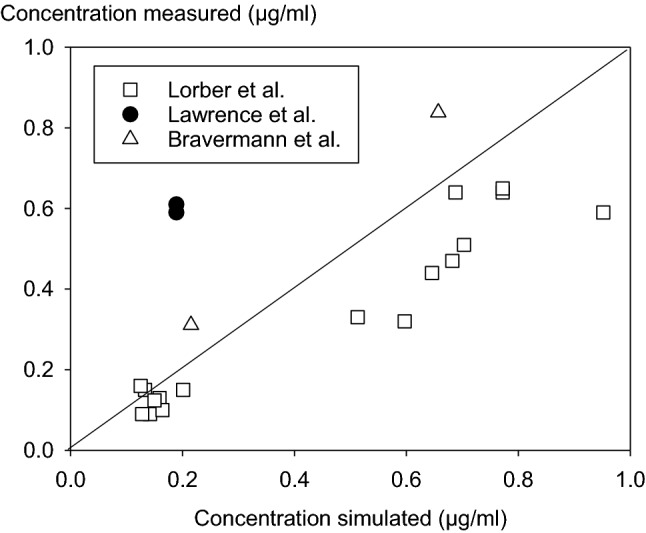
Measured serum perchlorate concentrations versus predicted concentrations using our model. Source of the measured data: Lawrence et al. (2000), Braverman et al. (2005), and Lorber (2009). Spearman correlation coefficient *R* = 0.737 (*p* = 0.000138)

**Fig. 3:**
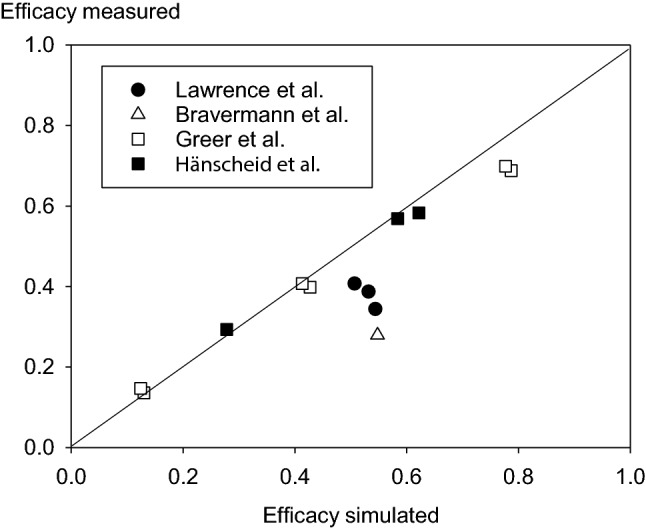
Protective efficacies derived from measured iodine-123 thyroidal uptake without and with perchlorate administration or exposure versus protective efficacies predicted by our model. Source of the measured data: Lawrence et al. (2000), Greer et al. (2002), and Braverman et al. (2005). Spearman correlation coefficient *R* = 0.909 (*p* = 0.00027)

The perchlorate concentration in serum predicted by our model correlated with the measured values of the considered studies (*r* = 0.737, *p* = 0.000138) (Fig. 2, Table 1). The protective efficacies against thyroidal iodine-131 uptake predicted for different perchlorate dosages by our model using a Km of 1.5 µmol l^−1^ differed only by a few % (range −15.7% to + 14.4%) and were on the average only slightly higher than the values measured in the experiments on volunteers described by Greer et al. (2002) and Hänscheid et al. (2011) (Fig. 3, Table 2). In both studies, the time of oral perchlorate intakes was clearly defined and could, therefore, be precisely taken into account in our simulations. Differences between our predicted values and the findings of Lawrence et al. (2000) or Braverman et al. (2005) were much larger (Fig. 3, Table 2). This may be due to the different study designs (e.g., drinking 1 l water with 10 mg perchlorate per day) (Lawrence et al. 2000) or exposure uncertainties at the working place (median absorbed dose 0.33 mg/kg shift) (Braverman et al. 2005).

**Table 1:** Measured serum perchlorate concentrations reported in the literature and values predicted when entering the respective perchlorate dosage schemes into our model

Dose	Measured (µg/ml)	Simulated (µg/ml)
Lorber (2009)		
0.5 mg kg^−1^ day^−1^	0.47	0.682
	0.64	0.688
	0.44	0.646
	0.59	0.952
	0.64	0.772
	0.33	0.513
	0.32	0.597
	0.65	0.772
	0.510	0.703
0.1 mg kg^−1^ day^−1^	0.15	0.133
	0.09	0.142
	0.09	0.128
	0.13	0.159
	0.16	0.125
	0.15	0.201
	0.1	0.164
	0.124	0.150
Lawrence et al. (2000)		
10 mg day^−1^ after 7 days	0.61	0.189
10 mg day^−1^ after 14 days	0.59	0.189
Braverman et al. (2005)		
0.33 mg kg^−1^ and shift intermediate	0.311 (mean)/0.153 (median)	0.215
During exposure	0.838 (mean)/0.359 (median)	0.657

**Table 2:** Measured radioiodine (I-123) uptake fraction from the literature and fractions calculated when entering the respective iodine and perchlorate dosage schemes into our model

Source	Measured I-123 uptake			Calculated I-123 uptake				
	Untreated	Perchlorate treated	Perchlorate efficacy	Untreated	Perchlorate treated (Km: 0.59 µM)	Perchlorate efficacy	Perchlorate treated (Km: 1.5 µM)	Perchlorate efficacy
Lawrence et al. (2000)	12.5% (4 h)	8.2%	0.344	10.09%	2.50%	0.752	4.60%	0.544
	17.3% (8 h)	10.6%	0.387	15.23%	3.92%	0.743	7.13%	0.532
	23.6% (24 h)	14%	0.407	20.23%	5.59%	0.724	9.97%	0.507
Braverman et al. (2005)	21.5%	15.5%	0.279	18.64%	2.80%	0.850	8.42%	0.548
Greer et al. (2002)	14.1% (8 h)	4.4% (0.5 mg/kg day)	0.688	15.23%	1.47%	0.904	3.24%	0.787
	21.6% (24 h)	6.5% (0.5 mg/kg day)	0.699	20.23%	2.06%	0.898	4.52%	0.777
	12.8% (8 h)	7.7% (0.1 mg/kg day)	0.398	15.23%	5.28%	0.653	8.73%	0.427
	19.9% (24 h)	11.8% (0.1 mg/kg day)	0.407	20.23%	7.29%	0.639	11.88%	0.413
	11.8% (8 h)	10.2% (0.02 mg/kg day)	0.136	15.23%	11.04%	0.275	13.25%	0.130
	18.4% (24 h)	15.7% (0.02 mg/kg day)	0.147	20.23%	14.89%	0.264	17.72%	0.124
Hänscheid et al. (2011)	24.8% (mean)	Not explicitly given	0.569 (100 mg at 2.1 h)	17.97% (mean 6,24,48 h)	6.64% (mean 6,24,48 h)	0.617 (mean 6,24,48 h)	7.27% (mean 6,24,48 h)	0.584 (mean 6,24,48 h)
	24.8% (mean)	Not explicitly given	0.583 (1000 mg at 2.2 h)	17.97% (mean 6,24,48 h)	6.44% (mean 6,24,48 h)	0.626 (mean 6,24,48 h)	6.52% (mean 6,24,48 h)	0.622 (mean 6,24,48 h)
	24.8% (mean)	Not explicitly given	0.293 (1000 mg at 7.4 h)	20.41% (mean 24,48 h)	14.68% (mean 24,48 h)	0.280 (mean 24,48 h)	14.72% (mean 24,48 h)	0.278 (mean 24,48 h)

Efficacy = 1—(iodine uptake with perchlorate/uptake without perchlorate)

Using as affinity constant of perchlorate for the NI-symporter, the lower *K*_*m*_ value of 0.59 µmol l^−1^ led to predicted efficacies around 20% larger (Table 2). As this revised lower *K*_*m*_ value is recommended only for exposures up to 100 µg kg^−1^ day^−1^ (Schlosser 2016) and predicted efficacies fit the measured values less, further simulations were performed using a *K*_*m*_ value of 1.5 µmol l^−1^.

### Comparison of the median protective doses of perchlorate and stable iodine after acute iodine-131 exposure

**Fig. 4:**
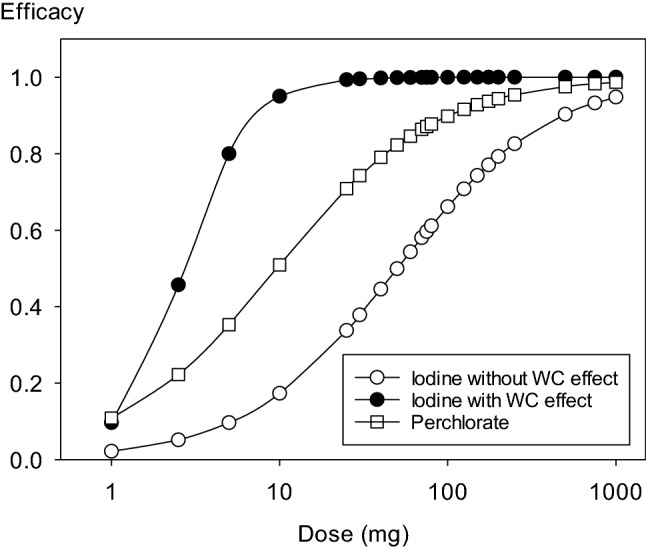
Dose efficacy curves for different single doses of perchlorate or stable iodine administered simultaneously with an acute iodine-131 exposure. Protective efficacy is based on the amounts of iodine-131 accumulated up to 24 h after exposure. Efficacy = 1—(thyroid dose with blockade/thyroid dose without blockade). WC effect: Wolff–Chaikoff effect (assumed duration at least 24 h)

With increasing single doses of perchlorate administered simultaneously to acute iodine-131 exposure, protective efficacy based on accumulated iodine-131 amounts in the gland 1 day after exposure increases up to about 1000 mg (potency ED_50_ = 9.60 mg for the reduction of thyroidal iodine-131 uptake after 24 h) (Fig. 4). The same applies to stable iodine if considering only the competition at the NI-symporter site. As expected from the higher Michaelis–Menten affinity constant, the mean effective dose of stable iodine is higher (ED_50_ = 50.11 mg). But taking into account the Wolff–Chaikoff effect, stable iodine shows a higher protective potency (ED_50_ = 2.70 mg) than perchlorate. Moreover, whereas the steepness of the linear portions of the dose–effect curves are comparable for perchlorate and stable iodine taking into account only the competition with iodine-131 at the NI-symporter (calculated Hill coefficients are 0.93 and 0.97, respectively), the slope is steeper for stable iodine if using the model including the Wolff–Chaikoff effect (Hill coefficient about 2.25) (Fig. 4). The results of our simulations confirm the finding that 1000 mg perchlorate roughly is equivalent to 100 mg stable iodine in its protective effect against a single acute radioiodine exposure (Hänscheid et al. 2011).

**Fig. 5:**
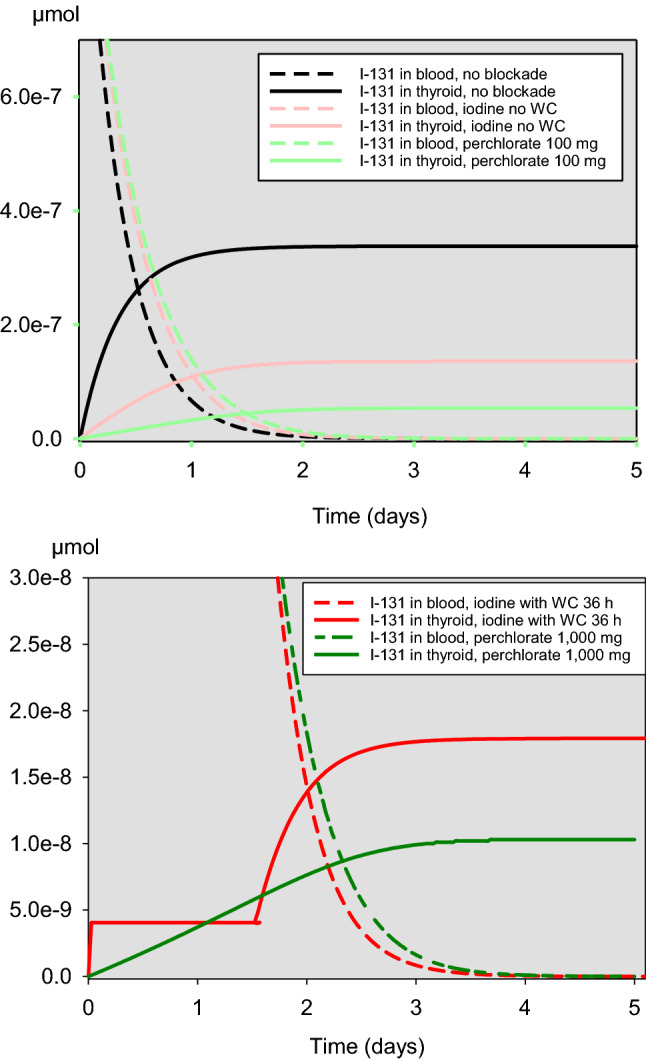
Time-course of the amount (µmol) of iodine-131 in blood/central compartment and the thyroid after acute exposure at *t* = 0 without or with thyroid blockade with iodine (100 mg) or perchlorate (100 mg or 1000 mg). WC: Wolff-Chaikoff effect lasting for 36 h

These findings are confirmed when considering the course of the accumulated iodine-131 in the gland over the time after acute exposure (Fig. 5). The curves describing the amount of iodine-131 in the gland after the administration of 1000 mg perchlorate or 100 mg iodine taking into account the Wolff–Chaikoff effect intersects roughly after 1 day, suggesting that for both interventions, protective efficacy is comparable. However, in the further course, the curves diverge before reaching plateaus at different levels on the third day, suggesting that 1000 mg perchlorate could confer a better protection than 100 mg iodine (Fig. 5). Although this is true numerically, taking into account the scaling and the distance to the values without blockade, efficacies 1 day and 5 days after acute exposure are quite comparable (iodine 0.987 and 0.947, perchlorate 1000 mg 0.988 and 0.970, respectively, for days 1 and 5).

### Estimation of protective efficacy of single perchlorate or iodine doses depending on the time of blockade initiation

**Fig. 6:**
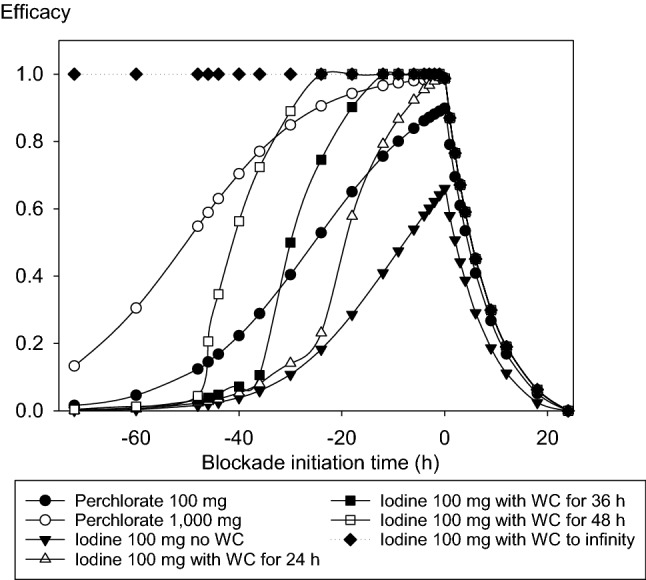
Protective efficacy of single doses of perchlorate or stable iodine (100 mg) depending on the time of administration before or after acute iodine-131 exposure at *t* = 0 h. Protective efficacy is based on the amounts of iodine-131 accumulated up to 24 h after exposure. Efficacy = 1—(thyroid dose with blockade/thyroid dose without blockade). *WC*: Wolff–Chaikoff effect. Calculations were performed for different durations of this effect corresponding to the range given in the literature (24–48 h). Results for an irreversible WC effect are not realistic and are shown only for illustration

Protective efficacy is maximal when perchlorate or stable iodine is administered simultaneously to acute iodine-131 exposure. The time–efficacy relations are not symmetrical in relation to the time point of acute iodine-131 exposure (Fig. 6). If thyroidal protection is initiated after acute iodine-131 exposure, efficacy decreases rapidly within hours for perchlorate 100 mg or 1000 mg as well as for stable iodine independently from the duration of the Wolff–Chaikoff effect, and there is practically no thyroidal protection against iodine-131 if the blockade is initiated after 24 h.

If administered prior to acute iodine-131 exposure, the protective efficacy of stable iodine heavily depends on the duration of the Wolff–Chaikoff effect (Fig. 6). If the blockade is for example initiated 30 h before acute iodine-131 exposure, the protective efficacy of a single stable iodine administration will amount to 0.141, 0.500, or 0.890 in case the Wolff–Chaikoff effect lasts 24 h, 36 h, or 48 h, respectively. If administered within 6 h before iodine-131 exposure, in all cases, protection exceeds 0.900. The longer the Wolff–Chaikoff effect is effective, the earlier stable iodine may be given before acute iodine-131 exposure without losing efficacy. An irreversible Wolff–Chaikoff effect lasting to infinity would theoretically guarantee a perfect protection independently of the time of administration prior to iodine-131 exposure, but this is not a realistic scenario (representation in Fig. 6 only for illustration).

For perchlorate, the loss of efficacy associated with an increasing duration between the time point of administration and iodine-131 exposure is less marked than for stable iodine (Fig. 6). If, for example, 1000 mg perchlorate or 100 mg iodine with a Wolff–Chaikoff effect lasting 48 h are administered 30 h before iodine-131 exposure, protective efficacy is in the same order of magnitude (0.849 vs. 0.890). Given 46 h prior exposure, perchlorate still achieves an efficacy of 0.589 in contrast to iodine with 0.206. Therefore, perchlorate at high doses may be of particular interest if the time of an expected iodine-131 exposure is difficult to predict.

### Protective efficacy of repetitive doses of perchlorate or stable iodine after acute iodine-131 exposure

**Fig. 7:**
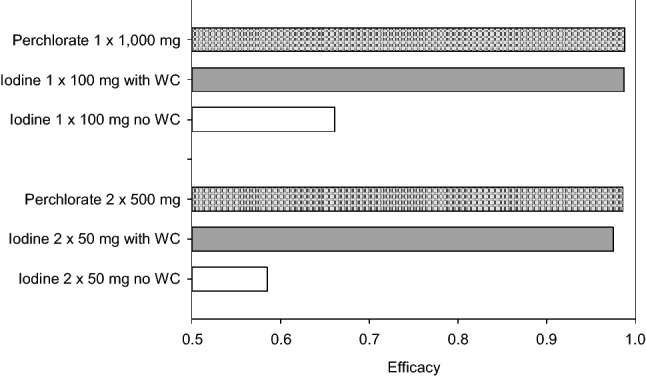
Protective efficacy of single or repetitive doses of perchlorate or iodine administered after acute iodine-131 exposure. The first dose is given at the time of exposure and the second dose if any after 12 h. Protective efficacy is based on the amounts of iodine-131 accumulated up to 24 h after exposure. Efficacy = 1—(thyroid dose with blockade/thyroid dose without blockade). *WC*: Wolff–Chaikoff effect (assumed duration at least 24 h)

Splitting the total dose of perchlorate to two identical smaller doses administered at shorter dosage intervals leads to a small numerical but not substantial loss of efficacy (e.g., efficacy 0.988 for 1 × 1000 mg vs. 0.986 for 2 × 500 mg) (Fig. 7, Table 3). In case of stable iodine, considering only the competition at the carrier site and without Wolff–Chaikoff effect, the reduction of efficacy if splitting the dose is more pronounced than for perchlorate (efficacy 0.661 for 1 × 100 mg iodine vs. 0.585 for 2 × 50 mg). Taking into account the Wolff–Chaikoff effect, splitting the dose into two equal smaller doses has only a marginal effect on efficacy as even the smaller individual doses cause a rapid total block of thyroidal iodine-131 uptake. Overall, the simulation results indicate that in case of acute iodine-131 exposure, it is not meaningful to split the available doses of perchlorate or iodine to permit repetitive administrations at short dosage intervals. The best protection is obtained by rapidly achieving high concentrations of perchlorate or stable iodine in blood at a time when iodine-131 concentrations are also high shortly after acute exposure.

**Table 3:** Protective efficacy of single or repetitive doses of perchlorate or iodine administered after acute iodine-131 exposure

Dose	Iodine without WC effect	Iodine with WC effect	Perchlorate
1 × 50 mg	0.499	0.975	0.820
2 × 50 mg	0.585	0.975	0.876
1 × 100 mg	0.661	0.987	0.898
2 × 100 mg	0.740	0.987	0.933
1 × 200 mg	0.793	0.994	0.945
1 × 500 mg	0.905	0.997	0.977
2 × 500 mg	0.936	0.997	0.986
1 × 1000 mg	0.950	0.999	0.988

The first dose is given at the time of exposure and the second dose if any after 12 h. Protective efficacy is based on the amounts of iodine-131 accumulated up to 24 h after exposure. Efficacy = 1—(thyroid dose with blockade/thyroid dose without blockade). *WC effect*: Wolff–Chaikoff effect (assumed duration at least 24 h)

### Protective efficacy of perchlorate or stable iodine in the case of continuous iodine-131 exposure

**Fig. 8:**
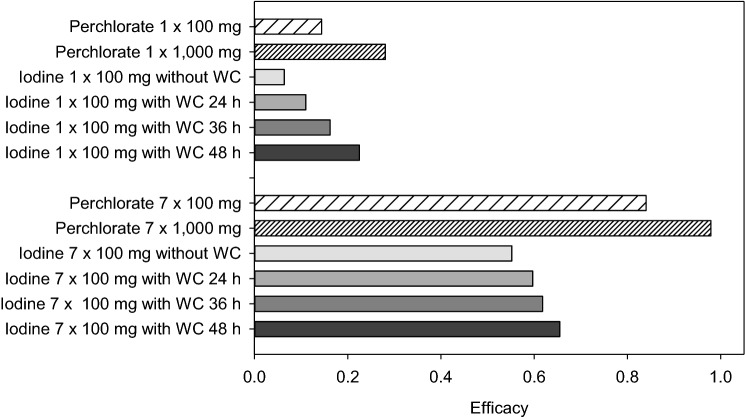
Protective efficacy of single or daily repetitive doses of perchlorate or iodine in the case of continuous iodine-131 exposure for 7 days. The first dose is given at the time exposure starts. Protective efficacy is based on the amounts of iodine-131 accumulated up to 24 h after the end of exposure (day 8). Efficacy = 1—(thyroid dose with blockade/thyroid dose without blockade). *WC*: Wolff–Chaikoff effect assumed to last 24–48 h after the thyroid gland is saturated with iodine

In the case of a continuous iodine-131 exposure, the protective efficacy of single doses of perchlorate or stable iodine, based on the total accumulated thyroidal iodine-131 24 h after the end of exposure (day 8) is much lower than for a scenario with acute exposure (e.g., for 1000 mg perchlorate 0.281 instead of 0.988) (compare Tables 3 and 4). Efficacy can be much improved by repetitive daily doses to block the gland (Fig. 8, Table 4).

**Table 4:** Protective efficacy of single or daily repetitive doses of perchlorate or iodine in the case of continuous iodine-131 exposure for 7 days

	Single dose	Repetitive daily doses for 7 days
Perchlorate 100 mg	0.144	0.840
Perchlorate 1000 mg	0.281	0.979
Iodine 100 mg without WC	0.064	0.552
Iodine 100 mg with WC 24 h	0.110	0.597
Iodine 100 mg with WC 36 h	0.162	0.618
Iodine 100 mg with WC 48 h	0.225	0.655

The first dose is given at the time exposure starts. Protective efficacy is based on the amounts of iodine-131 accumulated up to 24 h after the end of exposure (day 8). Efficacy = 1—(thyroid dose with blockade/thyroid dose without blockade). *WC*: Wolff–Chaikoff effect assumed to last 24–48 h after the thyroid gland is saturated with iodine

In the case of perchlorate, repetitive daily doses for the period of exposure of 7 days lead to almost the same protective efficacy as a single dose given simultaneously at acute iodine-131 exposure (e.g., efficacy for 1000 mg perchlorate 0.988 vs. 0.979). This is not the case for stable iodine. Protective efficacy of the latter increases with the duration of the Wolff–Chaikoff effect. But even if administered daily and assuming a Wolff–Chaikoff effect lasting 48 h, the protection of the gland is less than in the case of a single administration of stable iodine for an acute iodine-131 exposure (0.655 vs. 0.987) and against a continuous iodine-131 exposure, iodine blockade is clearly less effective than perchlorate according to our model (Fig. 8, Table 4).

**Fig. 9:**
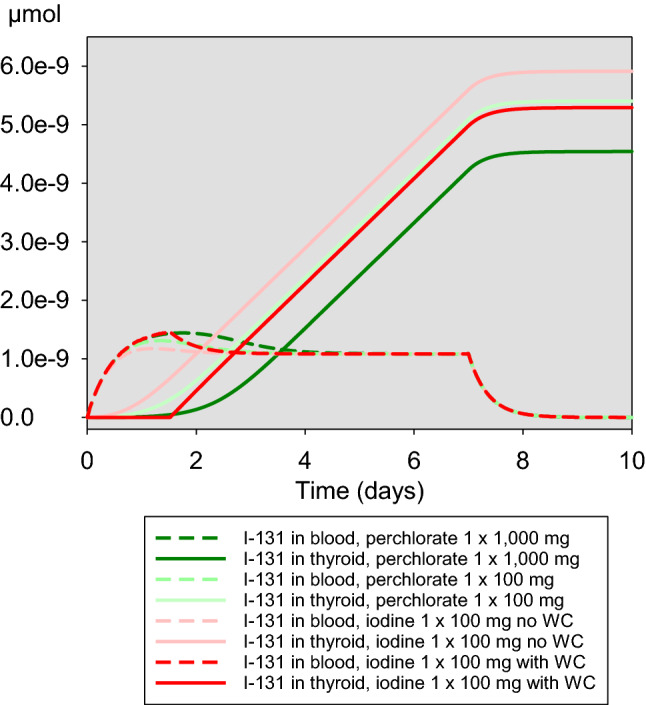
Time-course of the amount of iodine-131 in blood/central compartment and accumulated in the thyroid in the case of a continuous iodine-131 exposure of 7 days and a single administration of stable iodine or perchlorate at the time iodine-131 exposure starts. *WC* Wolff–Chaikoff effect lasting for 36 h

**Fig. 10:**
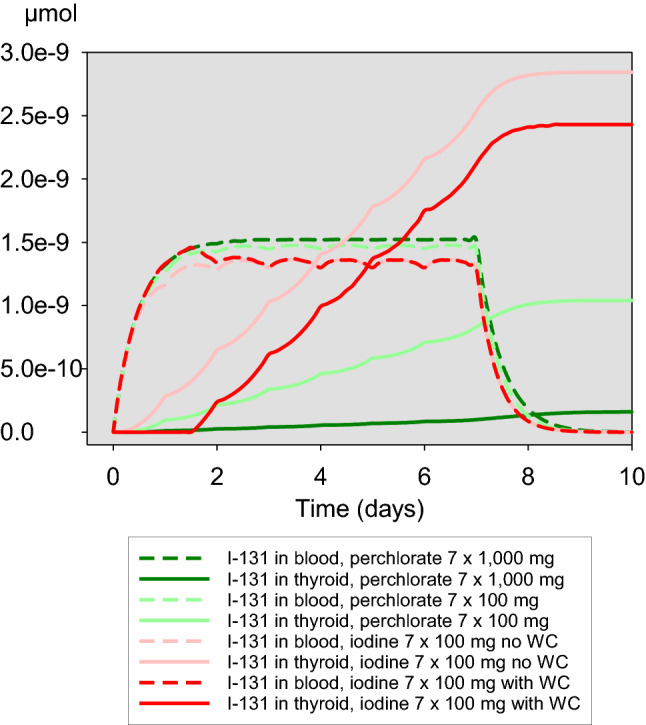
Time course of the amount of iodine-131 in blood/central compartment and accumulated in the thyroid in the case of a continuous iodine-131 exposure of 7 days and daily administrations of stable iodine or perchlorate for the whole exposure period. WC: Wolff–Chaikoff effect lasting for 36 h

This is supported when considering the course of the accumulated iodine-131 in the gland over time (Figs. 9, 10). Perchlorate 1000 mg is associated with the lowest thyroidal iodine-131 accumulation. Roughly, for the first 2 days after the beginning of the continuous iodine-131 exposure, taking into account the Wolff–Chaikoff effect, stable iodine seems to be slightly more effective than 100 mg perchlorate. If only a single dose of iodine or perchlorate (100 mg) is applied at the beginning of the exposure, the efficacy of both interventions is quite comparable in the further course (Fig. 9). In the case of repetitive daily administrations of 100 mg iodine or 100 mg perchlorate, the efficacy of the latter is clearly superior (Fig. 10).

## Discussion

The iodine transport at the basolateral membrane site of thyroidal cells is mediated by the active NI-symporter. Therefore, iodine blockade cannot be simulated with the original ICRP model using first-order kinetics, as the administration of 100 mg iodine will result in extracellular concentrations of iodide that cannot be considered as small compared to the Michaelis–Menten constant of the carrier (the initial concentration in the extracellular space after administration of 100 mg iodine amounts to 49 µmol l^−1^ vs *K*_*m*_ 9 µmol l^−1^). Besides an adequate structure, a valid compartment model requires correct parameters describing exchange processes between the compartments in man. For the iodine membrane carrier in mouse, rat and sheep thyroid *K*_*m*_ values of 20–40 µmol/l have been described (Rall et al. 1964) and it seems that for varying stimulations by TSH, the *K*_*m*_ values remain constant. The human NI-symporter has been cloned and it was shown that there is an 84% identity to the rat (Smanik et al. 1996). For the human iodine carrier in the thyroid, lower *K*_*m*_ values have been given (9 µmol/l) (Darrouzet et al. 2014) and that is the value we used in our model. It was previously shown that applying a thyroid model based on a carrier-mediated mechanism with our parameters to iodine-131 exposure alone leads to the same results as the equivalent doses computed with the established internal dosimetry software IMBA (Rump et al. 2019). For iodine blockade, the Wolff–Chaikoff effect inhibiting the hormonal synthesis and integration of iodine into thyroglobulin must be taken into account for a realistic modeling supported by empirical data (Wolff et al. 1969). This is problematic in so far as the precise mechanisms involved are not totally elucidated. We modeled the Wolff–Chaikoff effect by a saturation mechanism leading to a total, but only transient block of (radio)iodine uptake by the thyroid. As this effect is a physiological mechanism, it seems understandable that the iodine amounts needed to saturate the gland seems quite small [350 µg as reported by Ramsden et al. (1967)] compared to the mean daily iodine intake (about 150–250 µg/day) or the physiological uptake by the thyroid (about 70 µg/day) (Ramsden et al. 1967; De Groot et al. 1971). For modeling purposes, in our model, the Wolff–Chaikoff effect was artificially “switched on”, once the iodine saturation amount was reached, and “switched off” after 24–48 h. This is certainly a simplification, as it must be assumed that the activation and escape from the Wolff–Chaikoff effect is a progressive process. Nevertheless, our model combining competition at the carrier site and the Wolff–Chaikoff effect permits to well reproduce protective efficacies empirically measured after acute radioiodine exposure in volunteers (Rump et al. 2019), and, thus, seems to be useful for predictive purposes.

For perchlorate, the mechanisms involved for protection against radioiodine exposure seems less complex. Iodine organification is reported to remain unaffected (Wolff 1998), and thus, there is no mechanism comparable to the Wolff–Chaikoff effect to be taken into account. The Michaelis–Menten constant of the NI-symporter given for perchlorate has been given between 0.5 and 1.5 µmol l^−1^, with the lower values suitable only for low perchlorate concentrations, as expected in environmental conditions (Schlosser 2016). These values have been determined in vitro using Chinese hamster ovarian (CHO) cells in culture expressing the rat NI-symporter (Kosugi et al. 1996). Considering the substantial impact of this affinity constant on the results of protective efficacy calculations, the validation of our model using several empirical studies in man was of major importance. For our computations, we considered the inhibition of iodine transport by perchlorate at the NI-symporter, but at the difference of iodine, we did not take into account the transport of perchlorate itself into the thyroidal cells, although this is a known phenomenon. This would reveal difficult, as we have data neither on the maximum transport capacity through the membrane nor on an apparent rate constant describing the transport process as an approximate first-order kinetic in the case of very low perchlorate concentrations. Nevertheless, as we used pharmacokinetic parameters describing the elimination of perchlorate from the body represented as a single compartment without considering the thyroid, we can infer that perchlorate transport into the thyroid, if substantially affecting the disposition at all, is quantitatively included in the elimination rate, although renal elimination would be overestimated. The satisfactory comparability of the perchlorate concentration and protective efficacy values computed with the model and empirically measured, as reported in the literature, indicates that the model may be used for simulation and predictive purposes.

Thyroid blockade in the case of substantial radioiodine exposure is highly recommended (WHO 2017). The standard is the administration of stable iodine. According to the guidelines of the WHO, perchlorate is an alternative that may be considered in the case of iodine hypersensitivity (WHO 2017). The German Commission of Radiological Protection considers perchlorate to be a second choice alternative to iodine because of substantial adverse effects (SSK 2018). The official French guidelines do not mention perchlorate at all (ASN 2008). On the other side, based on experimental evidence in volunteers and adverse effects, the point of view that the choice of the agent used for thyroid blockade (iodine or perchlorate) is of minor importance was also expressed provided equieffective doses are administered (Hänscheid 2011). Perchlorate should be viewed as a relevant alternative to stable iodine in particular in patients with thyroid disorders and hyperfunction, considering the prevalence of this pathology (0.5–2% with a higher prevalence in women compared to men and increasing with age) (Garmendia Madariaga et al. 2014; De Leo et al. 2016; Journy et al. 2017). It should also be mentioned that the dose needed to achieve the same protective efficacy as 100 mg stable iodine (1000 mg perchlorate) is in a range that in Germany has an official approval for the initiation of hyperthyroidism treatment (Irenat® 300 mg perchlorate/ml = 15 drops; for adults 800–1000 mg/day in the first 1–2 weeks, in special cases up to 1500 mg/days, thereafter median daily dose 400 mg/days) or thyroidal protection in case of scintigraphy examinations of other organs using radioiodine (200–400 mg, in individual cases up to 1000 mg) or the perchlorate discharge test (600–1000 mg) (Gelbe Liste 2019). In the US, however, perchlorate is no longer available as the marketing of the medicinal product containing this substance (Perchloracap® with 200 mg perchlorate/capsule) has been discontinued (Reference.md 2020). In the US, perchlorate in drinking water and some foods is considered more as an environmental issue (Sellers et al. 2007; Maffini et al. 2016).

Our results confirm that thyroidal protection against iodine-131 exposure heavily depends on the exposure scenario. In the case of a longer lasting exposure, single doses of protective agents, whether stable iodine or perchlorate, offer substantially lower protection than after acute iodine-131 exposure, and thus, repetitive administrations seem necessary. In the case of a short-term acute iodine-131 exposure, iodine and perchlorate are similarly effective according to our model, provided that perchlorate is administered at a sufficiently high dose of 1000 mg. Although competition at the NIS carrier seems to be quantitatively the most important protection mechanism of high doses of stable iodine (66%), the Wolff–Chaikoff effect is required to reach a protective efficacy exceeding 90–95%. As this latter effect is fading after 24–48 h, it is quite understandable that stable iodine will lose effectiveness in case of a longer lasting continuous or repetitive iodine-131 exposure, even if administered repetitively. The higher affinity of perchlorate for the NIS carrier (9 µmol. l^−1^ for iodine, 1.5 µmol.l^−1^ for perchlorate) will confer the latter an efficacy advantage. Therefore, according to our model, stable iodine and perchlorate at equieffective dosages (100 mg iodine, 1000 mg perchlorate) are alternatives in case of short-term acute iodine-131 exposure, whereas preference should be given to perchlorate in view of its higher efficacy in the case of longer lasting iodine-131 exposures.

Although the loss of efficacy is rapid and quite similar for iodine and perchlorate if administered too late after an acute iodine-131 exposure, the protection of the gland is relatively well maintained according to our model even if perchlorate is given many hours too early before acute exposure, whereas the time point for iodine administration is more critical for efficacy. Thus, in the case, it is expected that optimal warning of the exposed population just in time may be difficult to achieve, the distribution of perchlorate and an official order to take the medication in doubt too early might be, certainly not the optimal, but the best achievable course of action.

Besides efficacy, the frequency and seriousness of side effects must be considered. Following the Chernobyl accident, a total of 17.5 million doses of potassium iodide were administered in Poland (children 10.5 million; adults 7 million) (Nauman 1993; Zarzycki 1994). A retrospective study was conducted among a group of 34,491 persons (12,641 children; 20,578 adults). The symptoms most frequently reported in questionnaires were vomiting, stomach ache, skin rashes, and headache. Two adults with known sensitivity to iodides are reported to have developed acute respiratory distress. Permanent thyroid dysfunction was not observed in more than 12,000 children who had received potassium iodide and only 12 of 3,214 examined newborns showed a transient thyroid inhibition at birth that resolved within 2–3 weeks (Nauman et al. 1993; 10). Overall, the percentage of adverse reactions to a single dose of potassium iodide was estimated at 0.2% (Jourdain et al. 2010).

As nuclear disasters are extremely rare events and a reliable retrospective documentation in such a large population as in Poland 1986 is difficult to realize, the data gathered must be evaluated with great caution and conclusions are also limited on the administration of a single iodine dose. Therefore, it seems prudent to take into account well-documented experiences from treatments with iodine or drugs containing and liberating iodine, e.g., amiodarone. High doses up to several hundred mg of iodine per day up to several weeks have been used for the treatment of Grave’s disease and/or prior to thyroidectomy (Calissendorf et al. 2017). Historically, Plummer used 80–320 mg daily for 10 days (Plummer 1924). A review of the literature indicates a low frequency or mild thyroidal and extrathyroidal adverse effects (Calissendorf et al. 2017; Suwansaksri et al. 2018). In patients treated with amiodarone, the incidence of the occurrence of thyroidal dysfunction (hyperthyroidism or hypothyroidism) is given with 2–12% (Trip et al. 1991). Hyperthyroidism is more frequently observed in iodine-deficient subjects or persons with multinodular thyroid glands with or without goiter, whereas patients with (euthyroid) autoimmune thyroiditis are particularly prone to develop hypothyroidism (Wolff 1998; Jourdain et al. 2010). However, amiodarone is a molecule that is metabolized at a very slow rate (terminal elimination half-live 30–90 days) (Holt et al. 1983; Plomp et al. 1984), so that the built-up of serum iodide concentration markedly differs from a single or even repetitive short-term potassium iodide administration. Moreover, it seems that amiodarone-induced thyroidal toxicity is mainly due to direct effects not related to iodine (Martino et al. 2001), as also suggested by ultrastructural changes different from those caused by excess iodine alone (Pitsiavas et al. 1997). The main metabolite, desethylamiodarone, has been reported to be even more cytotoxic than the parent compound (Beddows et al. 1989). Therefore, although amiodarone is often mentioned in relation with iodine toxicity, it is not a convincing comparison.

Perchlorate has been used for the treatment of hyperthyroidism with minor side effects like gastrointestinal irritations, rash, or lymphadenopathy that were found to be less than when using thionamide drugs (Krüskemper et al. 1960, 1962). However, adverse effects were reported to increase when dosages were increased from 600–1000 mg/day to 1500–2000 mg/day, so that recommended doses were set at 800 mg–1000 mg/day (Soldin et al. 2001). The use of perchlorate became very limited after the occurrence of seven cases of fatal aplastic anemia in the 60s whose pathophysiology is still unknown (Wolff 1998). Even a contamination of the badges was discussed as 4 of 7 cases occurred in a cluster. Although daily dosages were below 1000 mg, all patients had been treated for several months (Wolff 1998). It should be mentioned that thionamides (e.g., methimazole), another important class of antithyroid agents, are also known to cause serious potentially life-threatening side effects like agranulocytosis (Cooper 2005; Bukhari et al. 2017) or aplastic anemia (Escobar-Morreale et al. 1997; Yamamoto et al. 2004). Meanwhile, perchlorate is again effectively administered in the treatment of amiodarone-induced thyroid dysfunction, if necessary in addition to ethionamide drugs, and no serious side effects have been reported since this resurgence of use (Wolff 1998; Suwansaksri et al. 2018). Therefore, available data do not seem to preclude a short-term use of perchlorate for thyroidal protection against iodine-131 because of its toxicity.

A particularly sensitive population is pregnant women and newborns (Nishiyama et al. 2004; Reiners et al. 2013). Although it is clearly acknowledged that thyroidal protection against iodine-131 is particularly important in pregnant women and breastfeeding mothers (Verger et al. 2001; WHO 2017), it also is known that the capacity to escape from the Wolff-Chaikoff effect is less in immature glands. Even mild iodine overloads have been reported to cause fetal/neonatal hypothyroidism (Connelly et al. 2012; Sun et al. 2009; Jourdain et al. 2010). The transplacental transfer of thyroxine from the mother to the fetus beyond the first trimester is limited by placental deiodinases whose activities increase with gestation (Girling 2003). In cases of fetal thyroid dysfunction, it seems that placental deiodinases are inhibited and, moreover, the intracellular activation to triiodothyronine in the fetal brain activated, protecting the brain from permanent damage (Girling 2003). Nevertheless, it has also been reported that only transient hypothyroidism can impair the long-term mental development of the children or cause hearing impairments (Derksen-Lubsen et al. 1996; Kempers et al. 2006; Jourdain et al. 2010; Overcash et al. 2016), and screening at birth with postnatal treatment may not be sufficient to ascertain unimpaired neurodevelopment (Hardley et al. 2018). Therefore, the potential risks of side effects on the fetus resulting from iodine blockade should not be underestimated. This is reflected in the WHO recommendation that neonates, pregnant, and breastfeeding women should not receive repeated stable iodine doses due to the risk of adverse effects (WHO 2017). Therefore, in particular in the case of a continuous or repetitive iodine-131 exposure, it should be further analyzed whether perchlorate could be a reasonable alternative to stable iodine for thyroidal protection in this sensitive subpopulation, in view of its high efficacy and its simpler competitive action mechanism associated with rapid and safe reversibility when thyroid blockade is no longer needed.

## Conclusion

For cases of uncommon emergencies like nuclear or radiological incidents, real-life empirical data are sparse. Animal experiments give valuable information, but interspecies differences are associated with uncertainties. Experimental studies on volunteers are sometimes possible, but limited in scope for ethical reasons. Biokinetic and dosimetric models are difficult to validate and associated with uncertainties. Nevertheless, modeling and simulations may be an effective and efficient tool permitting to support planning processes and develop or optimize therapeutic dosage schemes. The findings based on our models of thyroidal protection by iodine and perchlorate seem to confirm the high efficacy of stable iodine in case of acute iodine-131 exposure, but show the superiority of perchlorate in the case of prolonged exposures. This deserves further analysis taking also into account the side effects and the sensitivity of particularly vulnerable subpopulations of patients. Moreover, in nuclear or radiological emergencies, prophylactic medications should never be considered as stand-alone protective actions, but should always be embedded in a broader plan for further protective measures like, e.g., sheltering and/or evacuation, even if this may reveal difficult when a larger number of victims are involved.
